# Supplementary material for: Interpretable brain disease classification and relevance-guided deep learning
Source: Sci Rep. 2022 Nov 24;12:20254. doi: 10.1038/s41598-022-24541-7 (PMC9691637; doi:10.1038/s41598-022-24541-7)
Supplement: Supplementary file 1 — Supplementary Information. [file 41598_2022_24541_MOESM1_ESM.pdf]

Interpretable Brain Disease Classification and Relevance-Guided Deep Learning

Christian Tinauer[1], Stefan Heber[1], Lukas Pirpamer[1], Anna Damulina[1], Reinhold Schmidt[1], Rudolf Stollberger[2,3], Stefan Ropele[1,3], and Christian Langkammer[1,3,*]

[1]Medical University of Graz, Department of Neurology, Graz, 8036, Austria
[2]Graz University of Technology, Institute of Biomedical Imaging, Graz, 8010, Austria
[3]BioTechMed-Graz, Graz, 8010, Austria
[*]christian.langkammer@medunigraz.at

Appendix A

Table A.1 shows search space and default values for the hyperparameter optimizations for all configurations.

**Hyperparameter**	**Default**	**Space**
learning rate	10^{-4}	$\log(10^{-5}, 10^{-3})$
γ of learning rate schedule	0.997	lin(0.96, 1.0)
β_1	0.9	-
β_2	0.999	-
batch size	8	-

Table A.1. Search space and default values for the hyperparameter optimizations for all configurations.

Appendix B

Table B.1 shows performance for the different models on all holdout data sets of cross validation.

Classifier	Skull stripping	Registration	Cross validation index	Balanced accuracy	Sensitivity	Specificity	AUC
CNN	no	-	1	73.28%	54.90%	91.67%	0.74
			2	67.02%	48.15%	85.90%	0.72
			3	70.19%	49.02%	91.36%	0.77
			4	70.41%	56.60%	84.21%	0.78
			5	75.39%	69.09%	81.69%	0.76
CNN	no	lin.	1	77.49%	64.71%	90.28%	0.76
			2	67.81%	57.41%	78.21%	0.75
			3	73.24%	58.82%	87.65%	0.84
			4	74.07%	54.72%	93.42%	0.80
			5	78.73%	80.00%	77.46%	0.87
CNN	no	nonlin.	1	83.62%	68.63%	98.61%	0.92
			2	71.94%	59.26%	84.62%	0.78
			3	75.93%	66.67%	85.19%	0.80
			4	74.64%	58.49%	90.79%	0.83
			5	81.93%	70.91%	92.96%	0.91
CNN	yes	-	1	80.15%	68.63%	91.67%	0.84
			2	70.66%	59.26%	82.05%	0.76
			3	78.36%	76.47%	80.25%	0.82
			4	75.50%	64.15%	86.84%	0.80
			5	83.66%	80.00%	87.32%	0.90
CNN	yes	lin.	1	78.35%	70.59%	86.11%	0.89
			2	77.14%	72.22%	82.05%	0.80
			3	76.98%	82.35%	71.60%	0.81
			4	78.81%	77.36%	80.26%	0.86
			5	85.98%	81.82%	90.14%	0.93
CNN	yes	nonlin.	1	89.09%	82.35%	95.83%	0.93
			2	74.07%	64.81%	83.33%	0.79
			3	79.99%	68.63%	91.36%	0.85
			4	81.99%	67.92%	96.05%	0.93
			5	85.48%	83.64%	87.32%	0.90
CNN+Graz$^+$	no	-	1	86.32%	82.35%	90.28%	0.92
			2	79.06%	72.22%	85.90%	0.88
			3	76.03%	70.59%	81.48%	0.81
			4	75.50%	64.15%	86.84%	0.84
			5	86.39%	85.45%	87.32%	0.93
CNN+Graz$^+$	no	lin.	1	92.44%	86.27%	98.61%	0.96
			2	89.67%	87.04%	92.31%	0.94
			3	75.82%	62.75%	88.89%	0.85
			4	83.22%	71.70%	94.74%	0.92
			5	89.82%	90.91%	88.73%	0.94
CNN+Graz$^+$	no	nonlin.	1	89.79%	82.35%	97.22%	0.93
			2	79.13%	68.52%	89.74%	0.89
			3	76.54%	66.67%	86.42%	0.84
			4	80.78%	77.36%	84.21%	0.88
			5	91.23%	90.91%	91.55%	0.94
Log. Regr.*	yes	lin.**	1	80.31%	74.51%	86.11%	0.89
			2	76.50%	72.22%	80.77%	0.86
			3	79.34%	78.43%	80.25%	0.85
			4	86.16%	86.79%	85.53%	0.94
			5	87.71%	90.91%	84.51%	0.95

Table B.1. Performance (in %) for the different models on all holdout data sets of cross validation.
*logistic regression by FSL-SIENAX (BET + tissue segmentation)
**linear registration is applied during FSL-SIENAX processing to obtain scaling factor
AUC, area under the curve of the receiver operating characteristics.
